# Evolution of Core Symptoms of Depression Disorders Among Chinese Adolescents Across Different Grades

**DOI:** 10.1155/da/2309327

**Published:** 2025-05-09

**Authors:** Xiaoxiao Song, Yaoyao Li, Xiaoyan Wang, Xindi Wang, Yanping Bao, Dianshun Zhang, Zhiqiang Li, Chenxia Meng, Changming Wang, Xiujun Zhang, Shaobo Lyu

**Affiliations:** ^1^Tangshan Key Laboratory of Mental Health and Cognitive Neuroscience, School of Psychology and Mental Health, North China University of Science and Technology, Hebei, China; ^2^Library, North China University of Science and Technology, Hebei, China; ^3^School of Basic Medical Health, North China University of Science and Technology, Hebei, China; ^4^National Institute on Drug Dependence and Beijing Key Laboratory of Drug Dependence Research, Beijing, China; ^5^Beijing Jingshan School Caofeidian Branch, Hebei, China; ^6^Tangshan Caofeidian Harbor Bussiness District Experimental School, Hebei, China

**Keywords:** adolescents, core symptoms, depression, gender differences, grade differences, network analysis

## Abstract

**Background:** Adolescence is a high-risk period for depression, especially after the COVID-19 pandemic, when adolescent depression has become increasingly severe. This study employs network analysis to identify core symptoms at various stages. It explores the differences in depression symptom characteristics among Chinese adolescents of different genders during elementary, middle, and high school periods.

**Methods:** A convenience sampling method was used to select 1553 students from various elementary, middle, and high schools in a specific city as participants. Their depression symptoms were assessed using the The Patient Health Questionnaire-9 (PHQ-9) depression screening scale. Using graph theory-based network analysis, this study constructs a depression symptom model via a correlation network and evaluates symptom nodes and their interconnections.

**Results:** The study found significant differences in the detection rates of depression symptoms among the three grade levels (*p*  < 0.001). However, no significant differences were found between male and female students in the detection rates and PHQ-9 scores (*p*  > 0.05). Through network analysis, this study identified the network changes in depression symptoms among Chinese adolescents of different grades and genders. The results show that “depressed mood” is the core symptom in the elementary and high school groups. At the same time, “fatigue” is the central factor affecting the depression network in the middle school group. Negative emotions and fatigue are the primary symptoms that run through the entire adolescent depression network.

**Conclusions:** This study reveals the heterogeneity of depression symptom networks among adolescent groups of different genders and grades, providing a theoretical basis for personalized interventions for adolescent depression in the future.

## 1. Introduction

The Diagnostic and Statistical Manual of Mental Disorders (DSM), published by the American Psychiatric Association, defines depression as a syndrome, which is a series of related symptoms that, in addition to negative emotions, are accompanied by behavioral characteristics such as withdrawal and distracted attention [1]. Short-lived feelings of sadness, disappointment, and frustration are normal in daily life. However, when these symptoms persist for weeks or months and cause discomfort in other areas (e.g., insomnia and loss of appetite), severely affecting an individual's social functioning, it may indicate major depressive disorder [2]. Nonclinical depressive symptoms and pathological depressive disorders differ in several aspects, including the emotional context, duration of symptoms, degree of social function impairment, accompanying physical symptoms, and biological rhythm changes [3].

During adolescence, the development of self-awareness is imbalanced, and stress-coping ability is weak. When faced with life events such as academic pressure, interpersonal relationships, and parent-child relationships, adolescents' negative coping styles and cognitive patterns can lead to depressive emotions [4, 5]. Increasing research indicates that depression is becoming more prevalent among younger individuals, especially after the COVID-19 pandemic [6]. The “2022 Adolescent Mental Health Status Report” pointed out that ~14.8% of Chinese adolescents are at risk of depression to varying degrees, with 4.0% of adolescents belonging to the high-risk group for severe depression and 10.8% to the mild depression risk group. Although there is a difference between nonclinical depressive symptoms and pathological depressive disorders [3], nonclinical depressive symptoms during adolescence do not fundamentally differ from pathological depressive disorders in causing maladaptation [7].

According to the network system perspective of depression [8, 9], depression is a network system composed of multiple nodes (depressive symptoms). These nodes interact with each other and influence each other. Analyzing this network system can identify key nodes (key symptoms), which helps to identify targets for intervention in depression, enabling precise prevention and treatment. Additionally, dynamics are also typical features of network systems, and by analyzing the patterns exhibited during the evolution of the network structure, one can further explore the changes in the network system and predict the development of the system. This dynamic feature of network systems can be utilized to anticipate sudden changes in depressive states at the individual level, enabling the development of tailored intervention or prevention plans [10].

Currently, most studies on adolescent depression use the overall score of depression measures as the unit of analysis. Few studies focus on the complex network structure of depressive symptoms and the differences in these structures across different genders and ages. This study examines children and adolescents, utilizing network analysis to identify the characteristics of the network model of depressive symptoms across different genders. It also aims to explore the dynamic characteristics of the depressive symptom network in this age group from a cross-sectional perspective.

## 2. Methods

### 2.1. Participants

Samples were drawn from multiple primary and secondary schools (including both regular and key schools) in the same city, covering students from diverse educational backgrounds. Gender ratios were balanced across all grades. Data from incomplete questionnaires or clearly invalid responses were excluded, resulting in a final valid sample of 1553 participants, including 883 elementary school students (grades 4–6) (*M* = 11.5, SD = 0.78), 358 middle school students (*M* = 14.1, SD = 0.32), and 312 high school students (*M* = 16.4, SD = 0.65). Among them, 805 were boys, and 748 were girls, with an overall average age of 12.4 years (SD = 2.58). All participants independently completed the questionnaire under guidance (Ethics approval number: 2024088).

### 2.2. Research Tools

#### 2.2.1. Depression Screening Scale (PHQ-9) [11]

This study adopted the nine criteria for depressive symptoms published by the American Psychiatric Association in the Diagnostic and Statistical Manual of Mental Disorders, which is a brief questionnaire designed to assess depressive symptoms. The questionnaire includes nine questions that address areas such as anhedonia, sad mood, sleep disturbances, fatigue, changes in appetite, guilt, difficulty concentrating, motor, and suicide. The questionnaire includes nine items, using a 4-point scale ranging from 0 to 3, with a total score ranging from 0 to 27. Scores of 0–4 indicate no depressive symptoms, 5–9 indicate mild depressive symptoms, 10–14 indicate moderate depressive symptoms, and 15 or above indicate severe depressive symptoms, with higher scores representing more severe depressive tendencies. The The Patient Health Questionnaire-9 (PHQ-9) scale is validated for depression screening among Chinese adolescents. Studies by Hu et al. [12] and Wei et al. [13] demonstrated robust psychometric properties in this population, including a Cronbach's alpha coefficient of 0.85, inter-item correlations ranging from 0.289 to 0.560, item-total correlations with the PHQ-9 total score between 0.616 and 0.730, a 4-week test–retest reliability of 0.88, and measurement invariance across gender and age groups. The scale had an alpha coefficient of 0.91 in this study.

### 2.3. Statistical Analysis

SPSS25.0 statistical software was used for descriptive statistics on PHQ-9 scores and correlations. Differences in detection rates were analyzed using the chi-square test, while group comparisons were conducted with independent samples *t*-tests and one-way ANOVA, followed by post hoc comparisons with Bonferroni correction. The Qgraph package in R software was used for network structure analysis and visualization, and correlation network models for depressive symptoms were constructed in the entire sample, the male and female groups. Nodes represent observed variables, and edges represent the partial correlation coefficients between two nodes after controlling for all other variables. The stronger the relationship between two nodes, the thicker and darker the edge is usually presented; the weaker the relationship, the thinner and lighter the edge.

The importance of each node is reflected by checking the node strength (strength) and node closeness (closeness) indicators, and the Bootstrap package was used to test the differences in the centrality of nodes. Since previous studies showed high stability of intensity centrality, this study mainly explained this centrality indicator and the other indicator was used as a reference [14]. In this study, all centrality coefficients are standardized *Z*-scores, so higher coefficients imply that the symptom is more likely to activate other symptoms in the network, thereby identifying the most important symptoms at various time points.

Network centrality testing was performed using the bootnet package in R software, with 1000 Bootstrap iterations [15]. The bootstrap method estimates the sample size for examining the stability of the centrality index, known as the correlation stability (CS) coefficient. A CS value greater than 0.25 indicates acceptable stability of node centrality.

The NetworkComparisonTest package was used to test the differences between networks in three stages. We performed three tests to assess the differences between networks: a network structure invariance test, a global strength invariance test, and an edge strength invariance test [16].

The study employed the powerly package for power analysis, utilizing the generalized Monte Carlo method. The sample size was estimated based on sensitivity, specificity, and correlation metrics [17].

## 3. Results

### 3.1. Power Analysis

The results indicated that when the sample size per symptom network exceeds *N* > 130, the network's correlation, sensitivity, and specificity reach acceptable thresholds (all above 0.6). The results of the power analysis are visualized in Figures S1–S3.

### 3.2. Detection Rate of Depressive Symptoms

Among the 1553 elementary and secondary school students, 660 were found to have depressive symptoms (PHQ-9 > 4), accounting for 42.5% of the total. Among these, 379 students had mild depressive symptoms (5–9 points), 177 students had moderate depressive symptoms (10–14 points), and 104 students had moderate to severe depressive symptoms (PHQ-9 ≥ 15).

### 3.3. Comparison of Depressive Symptoms Between Genders

There was no significant gender difference between the number of individuals identified with depressive tendencies (*p*  > 0.05), and no significant gender difference was observed in PHQ-9 scale scores (*p*  > 0.05). Additionally, no gender difference in the number of individuals identified or in scores was found across different grades (*p*  > 0.05) (Table 1).

### 3.4. Comparison of Depressive Symptoms Among Grade Groups

There was a significant difference in the number of students with depressive tendencies among the grade groups. Further multiple comparisons found significant differences in the detection rate of depressive symptoms between elementary and middle school students (*χ*2 = 48.413, *p*  < 0.001), between elementary and high school students (*χ*2 = 152.665, *p*  < 0.001), and between middle and high school students (*χ*2 = 24.936, *p*  < 0.001). There are also significant differences in PHQ-9 scores among the three grade groups (*p*  < 0.001). Post hoc multiple comparisons showed significant differences in scores between each pair of grades (*p*  < 0.001).

### 3.5. Network Analysis of Depressive Symptoms Across Grade Groups

**Figure 1:**
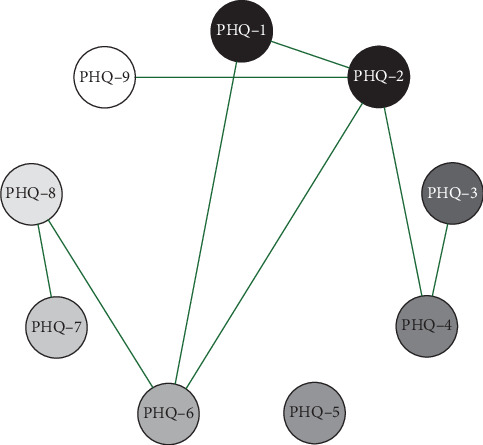
Primary School(Grades 4–6)Symptom Network Structure. Note; PHQ-1 Anhedonia; PHQ-2 Sad mood; PHQ-3 Sleep; PHQ-4 Fatigue; PHQ-5 Appetite; PHQ-6 Guilty; PHQ-7 Concentration; PHQ-8 Motor; PHQ-9 Suicide.

**Figure 2:**
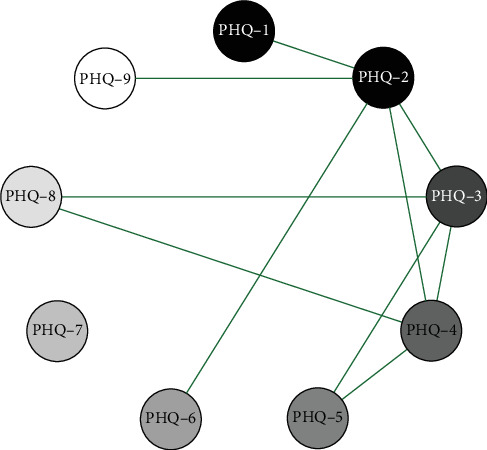
Middle school symptom network structure.

**Figure 3:**
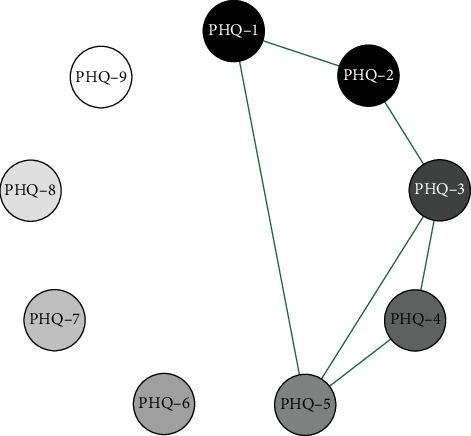
High school symptom network structure.

Network analysis results presented in Figures 1–3 show that in the elementary school group, the connection weight between item 3 (sleep disturbance) and item 4 (fatigue) on the PHQ-9 scale is the highest (0.78). In the middle school group, the connection weight between item 2 (sad mood) and item 4 (fatigue) is the highest (0.75). In the high school group, the connection weight between item 1 (anhedonia) and item 2 (sad mood) is the highest (0.75).

**Figure 4:**
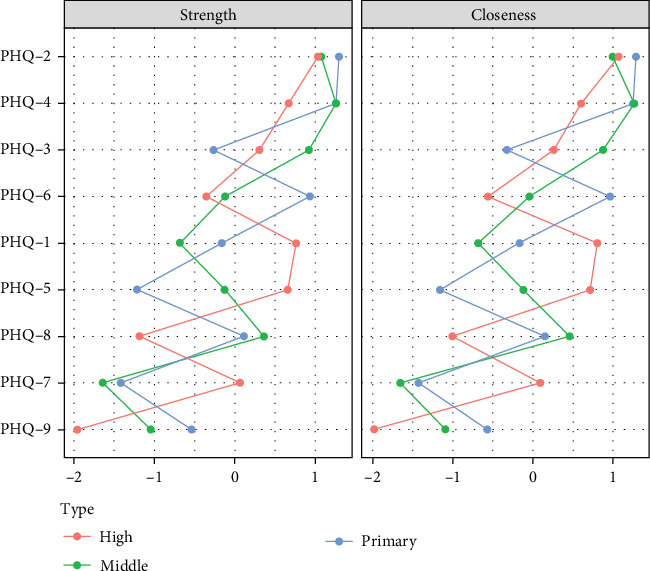
Network centrality indicators across different grade groups.

Centrality analysis (Figure 4) found that the second item (sad mood) in the primary school group had the highest intensity centrality. In the middle school group, the fourth symptom (fatigue) became the core symptom with the highest intensity centrality during this period. In the high school group, the second symptom (sad mood) became the symptom with the highest intensity centrality. Through network comparison, there were no significant differences (*p*  > 0.05) in network structure, global strength, and edge strength among the three grade groups. The stability test of network centralization measurement indicators found that the CS coefficient of strength centrality was 0.67, 0.44, and 0.52 in the three grade groups, respectively (Figures S4–S6).

### 3.6. Network Analysis of Depressive Symptoms by Gender Across Grade Groups

#### 3.6.1. Network Analysis of Gender Differences in Elementary School (Grades 4–6)

**Figure 5:**
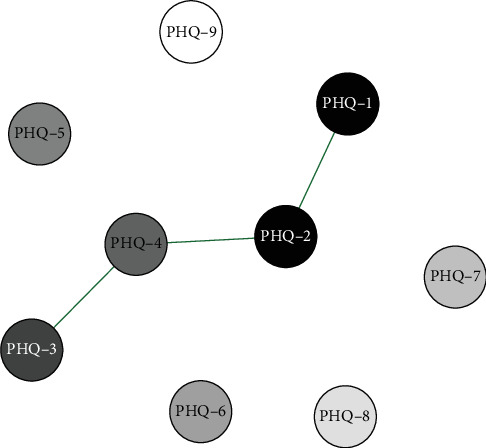
Primary school boys symptom network structure.

**Figure 6:**
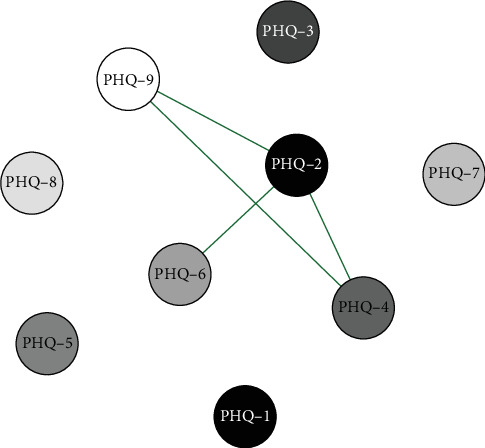
Primary school girls symptom network structure.

Network analysis results presented in Figures 5 and 6 show that in the elementary school boys group, the connection weight between item 4 (fatigue) and item 5 (appetite changes) on the PHQ-9 scale is the highest (0.90). In the elementary school girls group, the connection weight between item 4 (fatigue) and item 9 (suicide) is the highest (0.83).

**Figure 7:**
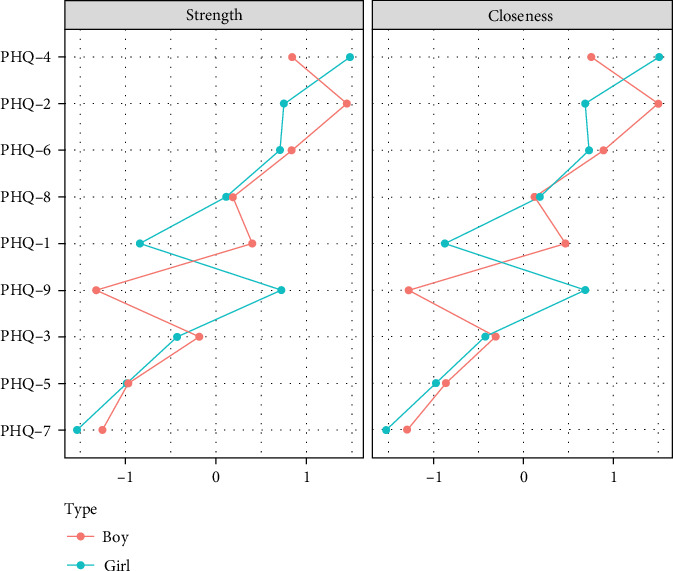
Network centrality indicators for primary school boys and girls.

Centrality analysis (Figure 7) revealed that the second item (sad mood) in the primary school boys' group exhibited higher intensity centrality. In the girls' group, the fourth item (fatigue) emerged as the core symptom with the highest intensity centrality during this period. Through network comparison, significant differences were observed in the network structure and global intensity between the two gender groups (*p*  < 0.01), with primary school girls exhibiting higher global intensity than boys. Edge weight comparison indicated significant differences in nine edges, all of which had higher weights for primary school girls than boys. Specifically, these edges included the fifth item, “appetite,” and the sixth item, “guilty” the fourth item, “fatigue,” and the seventh item,” concentration"; and edges 2, 3, 4, 5, 6, 7, and 8, each associated with the ninth item “suicide” (Table S1). Stability testing of network centrality measurement indicators revealed that the CS-coefficient of intensity centrality was 0.52 for both boys' and girls' groups (Figures S7 and S8).

#### 3.6.2. Network Analysis of Gender Differences in Middle School Students

**Figure 8:**
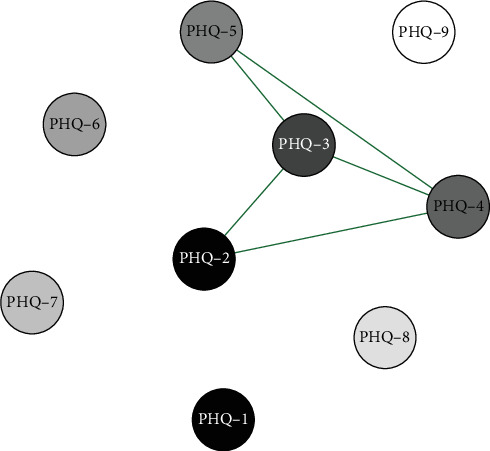
Middle school boys symptom network structure.

**Figure 9:**
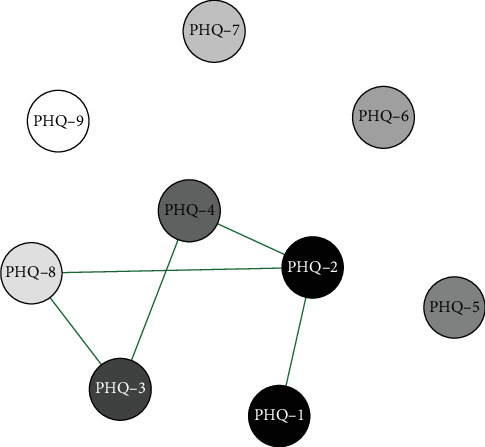
Middle school girls symptom network structure.

Network analysis results presented in Figures 8 and 9 show that in the middle school boys group, the connection weight between item 3 (sleep disturbance) and item 5 (appetite changes) on the PHQ-9 scale is the highest. In the middle school girls group, the connection weight between item 2 (sad mood) and item 4 (fatigue) is the highest (0.76).

**Figure 10:**
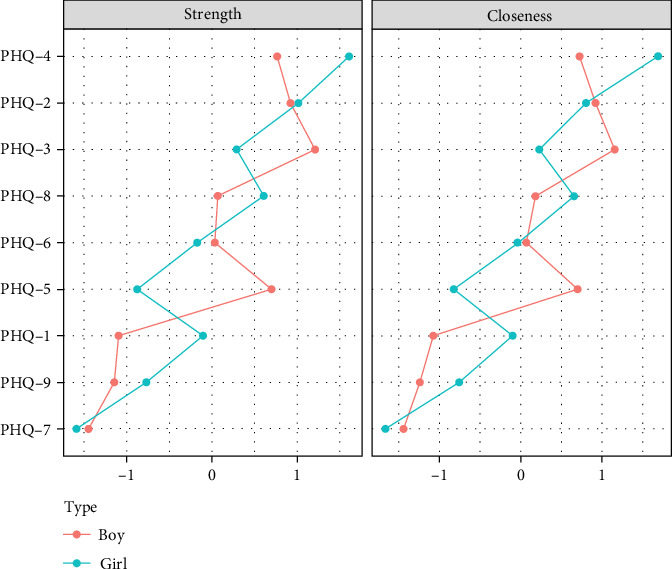
Network centrality indicators for middle school boys and girls.

Centrality analysis (Figure 10) revealed that the third item (sleep) in the middle school boys' group exhibited higher intensity centrality. In the girls' group, the fourth item (fatigue) emerged as the core symptom with the highest intensity centrality during this period. Through network comparison, no significant differences were observed in network structure, global strength, and edge weight among the three grade groups (*p*  > 0.05). Stability tests on network centrality measurement indicators indicated that the CS-coefficient of intensity centrality was 0.29 in both the boys' and girls' groups (Figures S9 and S10).

#### 3.6.3. Network Analysis of Gender Differences in High School Students

**Figure 11:**
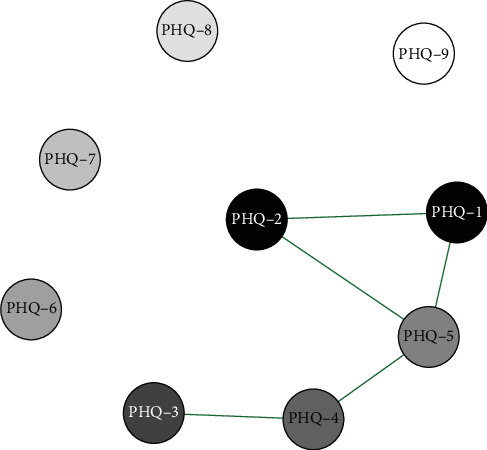
High school boys symptom network structure.

**Figure 12:**
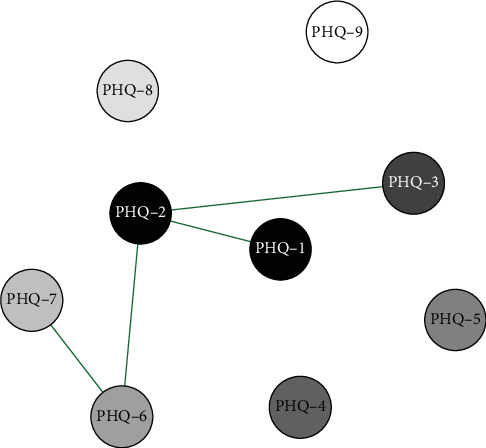
High school girls symptom network structure.

Network analysis results presented in Figures 11 and 12 show that in the high school boys group, the connection weight between item 4 (fatigue) and item 5 (appetite changes) on the PHQ-9 scale is the highest (0.90). In the high school girls group, the connection weight between item 6 (guilty) and item 7 (concentration) is the highest (0.67).

**Figure 13:**
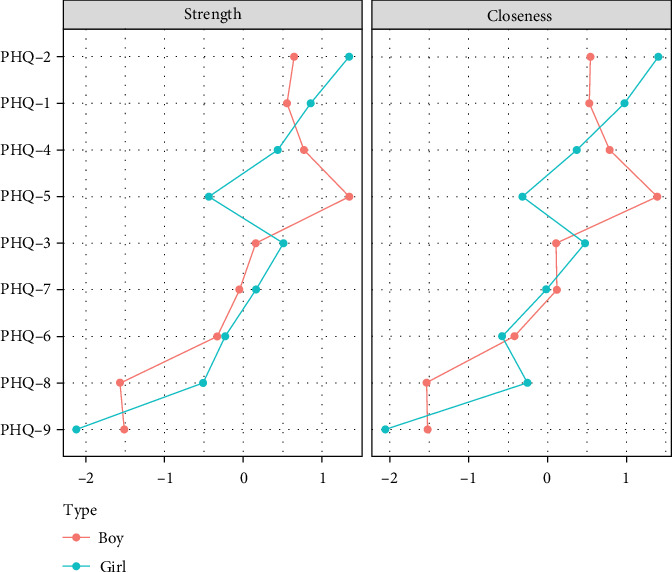
Network centrality indicators for high school boys and girls.

Centrality analysis (Figure 13) revealed that the fifth item (appetite) had a higher intensity centrality in the high school male group. In the female group, the second item (sad mood) emerged as the core symptom with the highest intensity centrality during this period. There were no significant differences in network structure between the two gender groups (*p*  > 0.05), but significant differences in global intensity were observed (*p*  < 0.01). Edge weight comparison indicated significant differences in four edges, with the weights of these four edges being higher in the high school male group than in the female group. Specifically, the first, second, fourth, and sixth items were significantly associated with the fifth item, “changes in appetite” (Table S2). Stability testing of network centrality measurement indicators revealed that the CS-coefficient of intensity centrality was 0.59 in the male group and 0.36 in the female group (Figures S11 and S12).

#### 3.6.4. Differences in Core Symptoms Between Boys and Girls Across Grades

**Figure 14:**
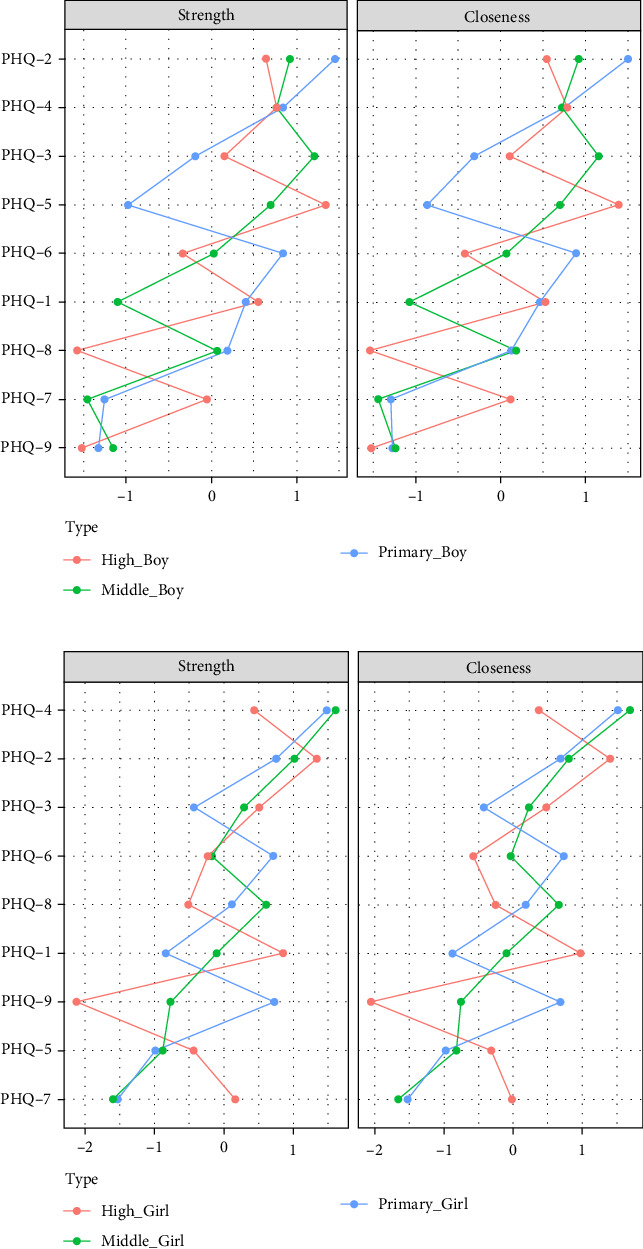
Centrality indicators of core symptoms across grades for boys and girls.

Figure 14 indicates that the symptom network structure did not differ significantly between primary and middle school male students (*p*  > 0.05), but showed a significant difference between primary and high school groups (*p*  < 0.01). The middle school and high school groups exhibited significantly higher network global strength than the primary school groups (*p*  < 0.01). However, there were no significant differences in network structure and global strength between the middle and high school groups. In terms of strength centrality, the “sad mood” symptom was significantly higher in the primary school group compared to the high school group (*p*  < 0.05), indicating a decline in the strength centrality of the second item (sad mood) with increasing grades. Meanwhile, the third item (sleep disturbance) was significantly higher in the middle school group compared to the primary school group (*p*  < 0.05), with no significant difference between the middle school and high school groups. The fifth item (changes in appetite) emerged as the most core symptom in the high school group (*p*  < 0.05).

Among female students in different grades, there were no significant differences in network structure and global strength between the primary and middle school groups (*p*  > 0.05). However, significant structural differences were observed compared to the high school group (*p*  < 0.01), with the network global strength of the primary school group being significantly higher than that of the high school group (*p*  < 0.01). There were no significant differences in network structure and global strength between the middle and high school groups. Item 4 (fatigue) and Item 9 exhibited the highest strength centrality in primary school (*p*  < 0.05), while other symptoms remained relatively stable.

## 4. Discussion

### 4.1. Discussion of Research Results

This study found no gender differences in the detection rate and scores of depressive symptoms among males and females in the entire sample, nor were there differences between the overall sample and various grade groups. Domestic research has also found that the incidence of depressive mood among adolescent boys is as high as that among girls [18]. Detection rates vary significantly among grade groups; high school students have a higher detection rate than middle school students, who, in turn, have a higher rate than primary school students. It is consistent with previous research findings, indicating that individual depressive symptoms begin to show a significant upward trend in early adolescence [2, 19]. Meanwhile, research involving Chinese adolescents as subjects has found that middle school is a critical grade for the development of adolescent depression, after which depressive symptoms begin to show a significant upward trend [20]. This study also found that the detection rate of severe depression among adolescents increases with each grade group. The early- and mid-adolescence period is a critical period for the development of depression, and it is of great significance to implement early intervention and treatment during this period.

### 4.2. Core Symptoms of Depression in Different Grade Groups

This study utilizes network analysis to compare the network changes in depressive symptoms among adolescents of different grades and genders, identifying core symptoms at various stages. The results confirm the heterogeneity of the network of depressive symptoms among adolescents of different grades. The network analysis indicates that the core symptoms of depressive tendencies among adolescents of different grades share both similarities and differences. Specifically, “moodiness” and “fatigue” exhibit high-intensity centrality across the three grade groups. Moodiness manifests as persistent sadness, disappointment, or hopelessness, while fatigue is often associated with physical and mental exhaustion. The students in this study are generally in adolescence, a rapid physiological and psychological development stage. Changes in sex and stress hormones (such as cortisol) may lead to emotional fluctuations and energy consumption, subsequently affecting mental states [21].

Additionally, increased academic difficulty and gradually emerging exam pressure may lead to a greater sense of fatigue [22]. Meanwhile, there is no difference in the overall network structure and intensity among the three grade groups, indicating that the network of depressive symptoms can remain unchanged and unaffected by age. Furthermore, the overall intensity does not significantly increase from early to late adolescence, suggesting that symptom connectivity remains consistent across the three grades. However, it is also noteworthy that, despite the similarity in core symptoms across the three grades, the most correlated symptoms vary among them. Specifically, in the primary school group, moodiness has the highest connection weight with sleep disorders (referring to changes in sleep patterns, which may manifest as difficulty falling asleep or excessive sleepiness). In the middle school group, it is moodiness and fatigue; in the senior high school group, it is loss of interest and moodiness. When the core symptoms of the depressive network are activated, they are likely to propagate activation and affect the entire network through other symptoms connected to them [23]. Therefore, paying attention to their correlated symptoms is also of great significance.

### 4.3. Differences in Core Symptoms of Depression Between Genders in Different Grade Groups

First, girls in primary school have a higher network intensity of depression compared to boys of the same age. According to the pathological network theory, an increase in global intensity means that when one symptom is activated, other symptoms are also more likely to be activated, leading to the deterioration of the disease [24]. Furthermore, through a comparison of edge weights, it is observed that the edges where girls have higher weights than boys are concentrated on suicide ideation and its connected nodes. This study also found that among girls in three different grades, those in primary school have the highest centrality in terms of suicide ideation node intensity. Some studies have pointed out that early adolescent girls have higher levels of negative emotions and suicide ideation compared to boys [25]. After entering puberty, rapid changes in hormone levels can exacerbate girls' negative experiences and increase the incidence of internalizing problems [22]. Research indicates that, compared to boys, girls are more likely to ruminate after experiencing negative life events. This tendency can result in heightened negative emotions and ineffective coping strategies [26]. Therefore, it is important to prioritize the mental health of girls and to implement timely and effective interventions.

Second, the intensity of depression in high school boys is higher than that of girls during the same period, with the difference in edge weights concentrated on changes in appetite and the nodes connected to it. Specifically, the weight of related edges for boys is significantly higher than for girls. Moreover, within the entire male group, the centrality of the intensity of appetite change increases with grade level, reaching its peak during high school. For high school boys, the symptom of “changes in appetite” requires attention due to the increase in adolescent depression and psychological stress. Boys may be more prone to emotional triggers than girls, choosing emotional eating, which includes higher cortisol response and higher intake of fats and sugars [27, 28]. Evidence suggests that highly processed foods (i.e., refined carbohydrates and foods high in fat) may trigger an addictive process, during which adolescents may be passively forced to eat [29].

Finally, within the male group, the global intensity of the depression network is higher during the middle and high school stages compared to elementary school. In contrast, the female group shows the opposite trend, with the global network intensity higher in elementary school than in high school. It may be related to boys' and girls' different brain development speeds. Depression symptoms are closely related to the development of brain regions responsible for emotional regulation. Generally, the prefrontal cortex, responsible for higher cognitive functions, is the last area to develop, maturing around age 25 [30]. It is commonly believed that emotional regulation involves the prefrontal cortex inhibiting the neural activity of the amygdala [31]. At the same time, throughout adolescence, the brain regions related to inhibitory functions gradually mature and become more activated [32]. Since adolescent girls' brains mature earlier than boys' [33], this leads to different trends in the changes in depression network intensity between adolescent boys and girls. Furthermore, “sad mood” is a core symptom in the depression networks of all three grade groups. However, its centrality decreases in the male group as grade level increases, which may also be related to brain development.

In conclusion, this study used cross-sectional network analysis to reveal that the core symptoms of depression among Chinese adolescents evolve with age and changes in the environment. It also reflects that different stages and genders of adolescent groups may have different trajectories of depression development [34]. The results provide a theoretical basis for researchers to develop specific interventions for depressive symptoms in adolescents at different stages.

### 4.4. Limitations and Future Directions

This study also has some limitations. First, the sample was drawn from middle and primary schools in a specific urban area, which limits the sample's representativeness. Future research could employ a systematic stratified sampling method to broaden the sample coverage, ensuring the inclusion of adolescents from various age groups and backgrounds. This approach would help verify and extend the applicability of the results.

Additionally, while cross-sectional network analysis can reveal correlations and dependencies between variables, it has limitations in explaining the psychological mechanisms and reasons behind these relationships. It cannot fully capture the changes in a single set of symptoms over time. Therefore, to gain a more comprehensive understanding of the development trajectory of adolescent depression, longitudinal studies should be incorporated for in-depth exploration. Future research could design longer-term tracking studies, which would help to examine the validity of the network model more deeply.

## 5. Conclusion

“Sad mood” is the core symptom in the elementary and high school groups across the entire network. At the same time, “fatigue” is the central factor affecting the depression network in the middle school group.

Negative emotions and fatigue are the main symptoms that permeate the entire adolescent depression network.

## Figures and Tables

**Table 1:** Comparison of differences in depressive symptoms across gender and grade.

Variable	*n*	Depressive symptoms [*n* (%)]		*χ* ^2^	*M* ± SD	*t*	*F*
		None	Present				
Gender (Total)	—	—	—	2.092	—	−0.326	—
Male	805	466 (57.9)	339 (42.1)	—	5.03 ± 5.49	—	—
Female	748	427 (57.1)	321 (42.9)	—	5.12 ± 5.48	—	—
Primary school	—	—	—	2.239	—	0.469	—
Male	468	319 (68.1)	149 (31.8)	—	3.70 ± 4.43	—	—
Female	415	302 (72.8)	113 (27.2)	—	3.55 ± 4.97	—	—
Middle school	—	—	—	0.548	—	0.018	—
Male	179	92 (51.4)	87 (48.6)	—	5.96 ± 6.17	—	—
Female	179	85 (47.5)	94 (52.5)	—	5.95 ± 5.23	—	—
High school	—	—	—	2.875	—	−0.711	—
Male	158	55 (34.8)	103 (65.2)	—	7.92 ± 6.19	—	—
Female	154	40 (26.0)	114 (74.0)	—	8.39 ± 5.47	—	—
Grade	—	—	—	162.406*⁣*^*∗∗∗*^	—	—	94.946*⁣*^*∗∗∗*^
Primary school	883	621 (70.3)	262 (29.7)	—	3.63 ± 4.69	—	—
Middle school	358	177 (49.4)	181 (50.6)	—	5.96 ± 5.71	—	—
High school	312	95 (30.4)	217 (69.6)	—	8.16 ± 5.84	—	—

*⁣* ^*∗∗∗*^ *p*  < 0.001.

## Data Availability

The data that support the findings of this study are available from the corresponding author upon reasonable request.

## Supporting Information

Supporting Information Figure S1. Specificity index results. Figure S2. Sensitivity index results. Figure S3. Correlation index results. Figure S4. Centrality stability for depressive symptoms network in the primary school sample. Figure S5. Centrality stability for depressive symptoms network in the middle school sample. Figure S6. Centrality stability for depressive symptoms network in the high school sample. Figure S7. Centrality stability for depressive symptoms network in the primary school boys. Figure S8. Centrality stability for depressive symptoms network in primary school girls. Figure S9. Centrality stability for depressive symptoms network in the middle school boys. Figure S10. Centrality stability for depressive symptoms network in middle school girls. Figure S11. Centrality stability for depressive symptoms network in the high school boys. Figure S12. Centrality stability for depressive symptoms network in the high school girls. Table S1. Edge invariance test between primary school boys and girls (*p* < 0.05). Table S2. Edge invariance test between high school boys and girls (*p* < 0.05).
