# Supplementary material for: Evolution of Core Symptoms of Depression Disorders Among Chinese Adolescents Across Different Grades
Source: Depress Anxiety. 2025 May 9;2025:2309327. doi: 10.1155/da/2309327 (PMC12084781; doi:10.1155/da/2309327)
Supplement: Supporting Information — Figure S1. Specificity index results. Figure S2. Sensitivity index results. Figure S3. Correlation index results. Figure S4. Centrality stability for depressive symptoms network in the primary school sample. Figure S5. Centrality stability for depressive symptoms network in the middle school sample. Figure S6. Centrality stability for depressive symptoms network in the high school sample. Figure S7. Centrality stability for depressive symptoms network in the primary school boys. Figure S8. Centrality stability for depressive symptoms network in primary school girls. Figure S9. Centrality stability for depressive symptoms network in the middle school boys. Figure S10. Centrality stability for depressive symptoms network in middle school girls. Figure S11. Centrality stability for depressive symptoms network in the high school boys. Figure S12. Centrality stability for depressive symptoms network in the high school girls. Table S1. Edge invariance test between primary school boys and girls (p < 0.05). Table S2. Edge invariance test between high school boys and girls (p < 0.05). [file 2309327.f1.docx]

**Supporting Information**

**1. Power analysis**

**
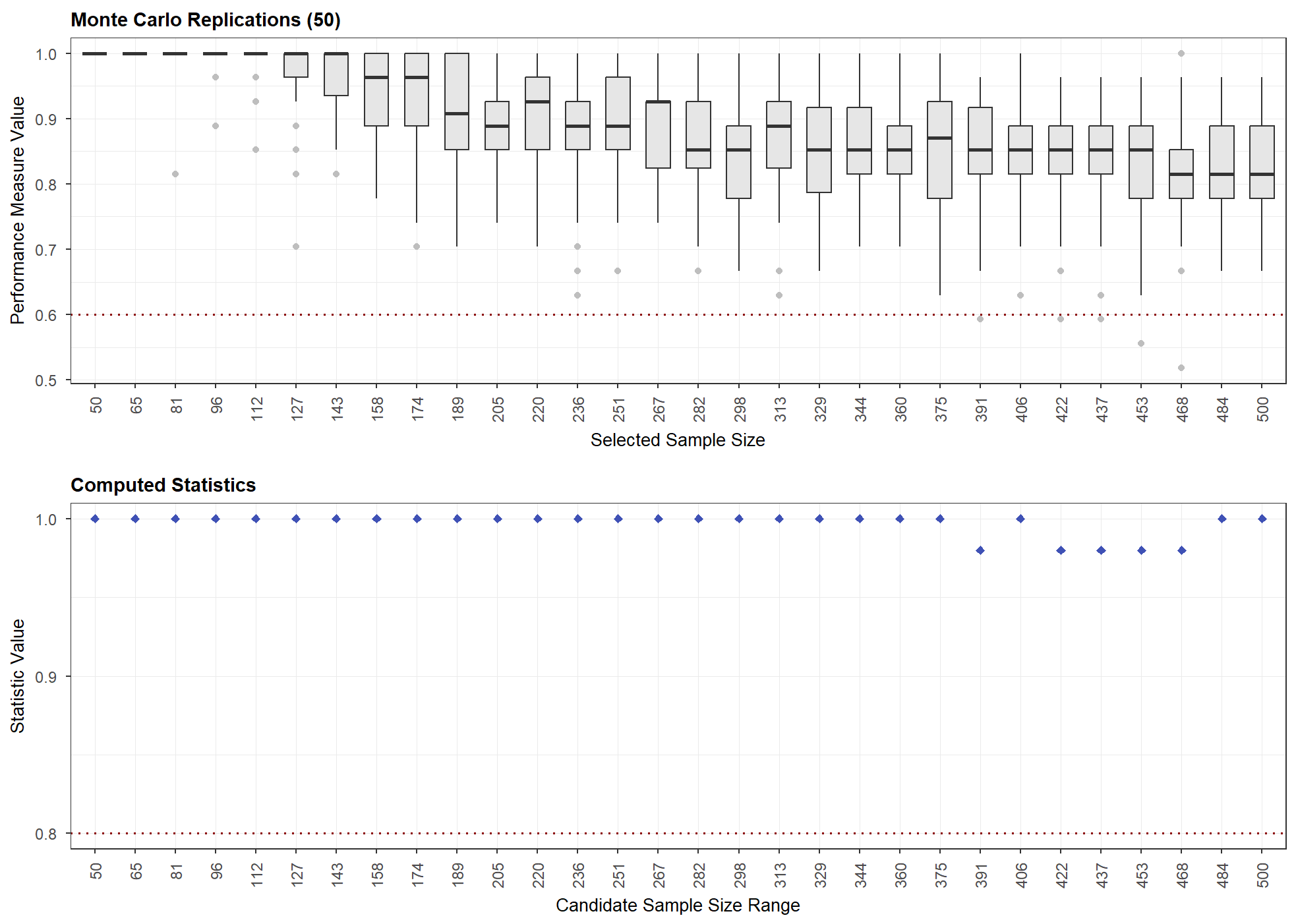
**

**Figure S1. Specificity index results**

**
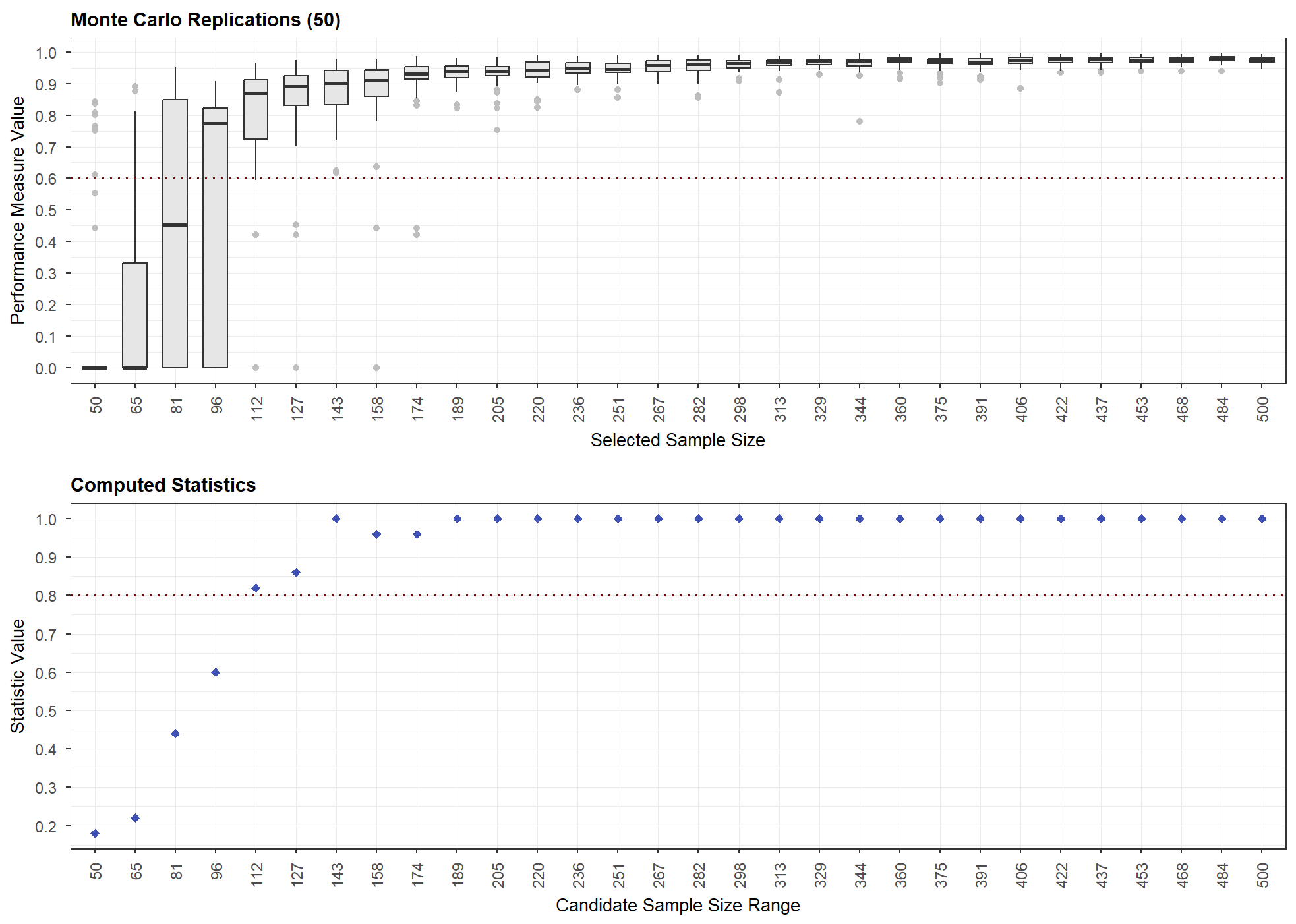
**

**Figure S2. Sensitivity index results**

**
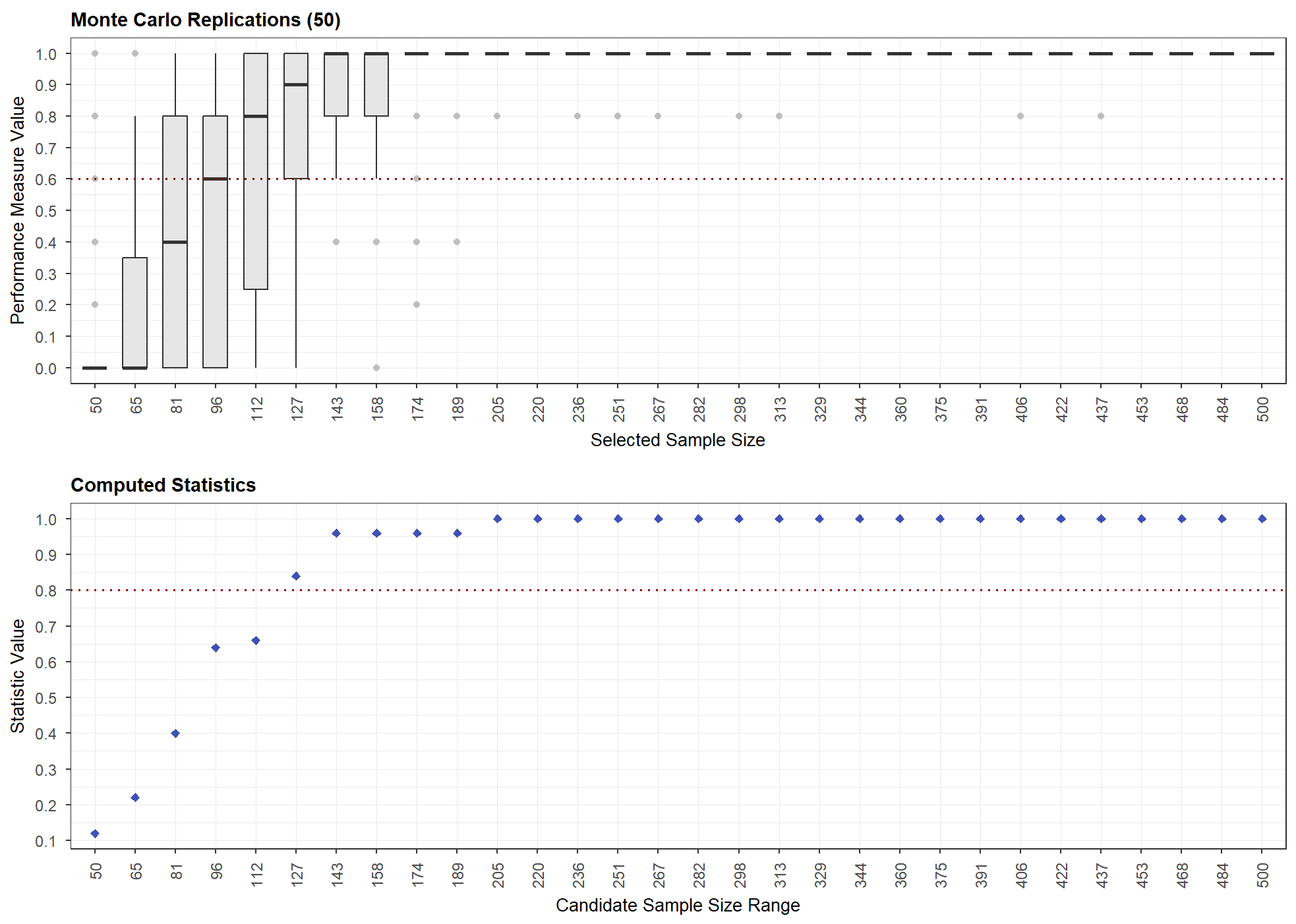
**

**Figure S3. Correlation index results**

**2. Regarding the stability results (CS) of the three-grade group networks**

Average correlations between centrality indices of networks sampled with persons dropped and the original sample. Lines indicate the means, and areas indicate the range from the 2.5th quantile to the 97.5th quantile.


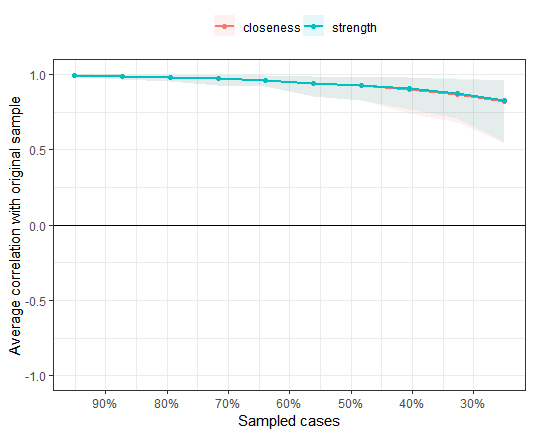


**Figure S4.** Centrality stability for depressive symptoms network in the primary school sample.

**
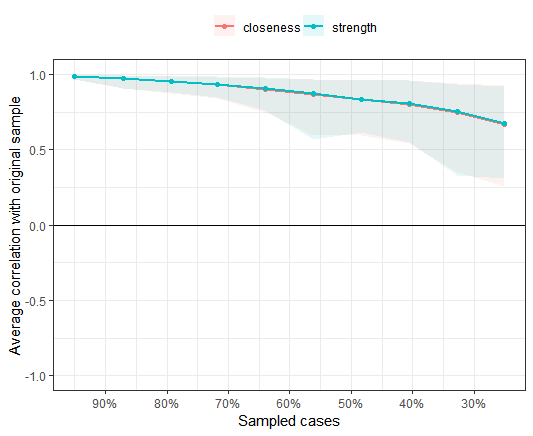
**

**Figure S5.** Centrality stability for depressive symptoms network in the middle school sample.

**
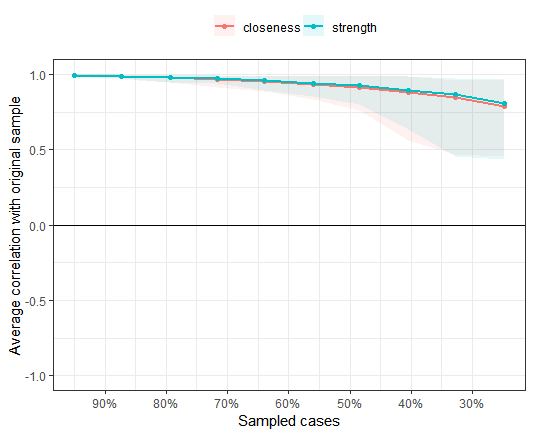
**

**Figure S6.** Centrality stability for depressive symptoms network in the high school sample.


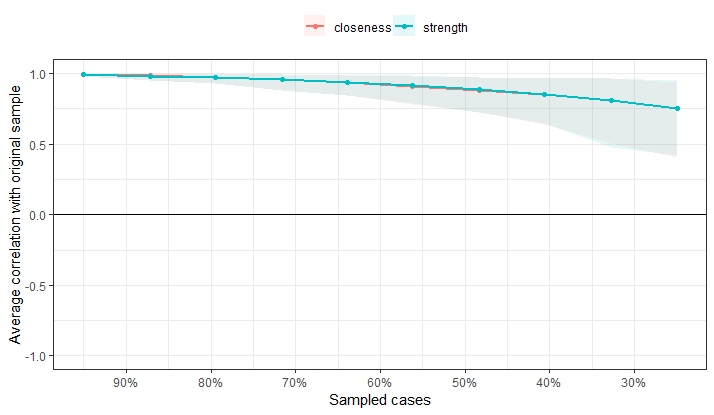


**Figure S7.** Centrality stability for depressive symptoms network in the primary school boys.


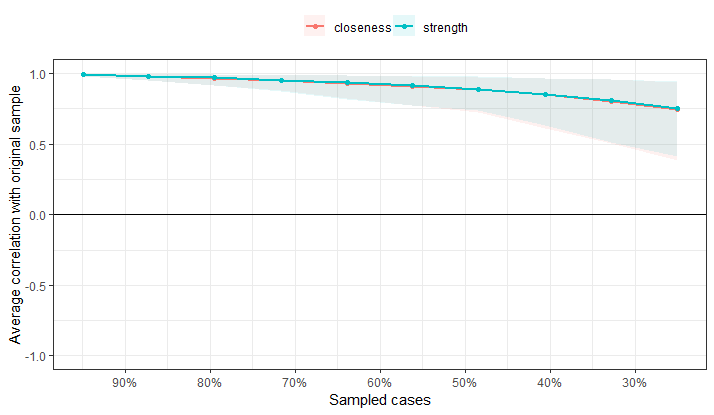


**Figure S8.** Centrality stability for depressive symptoms network in primary school girls.


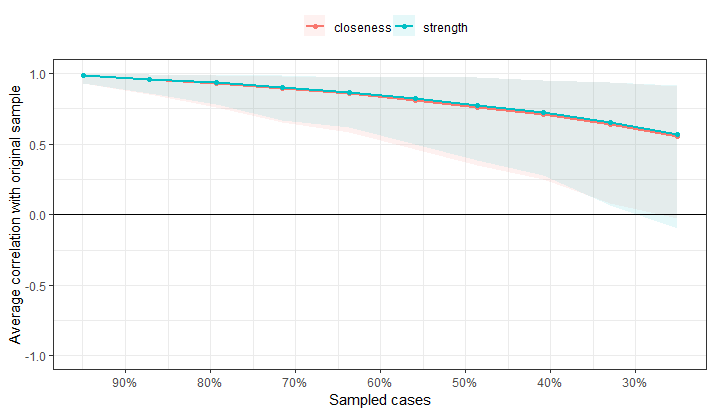


**Figure S9.** Centrality stability for depressive symptoms network in the middle school boys.


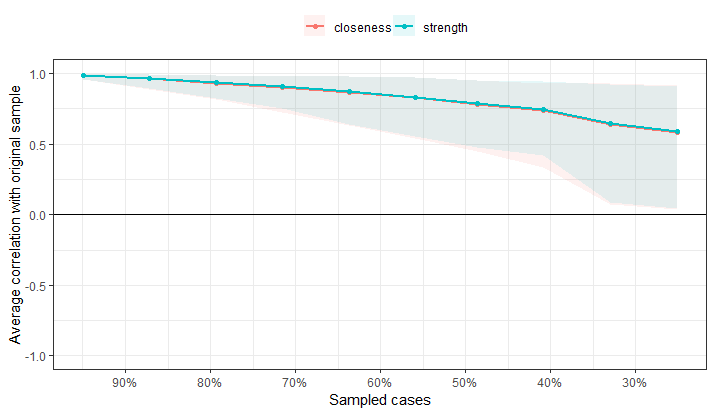


**Figure S10.** Centrality stability for depressive symptoms network in middle school girls.


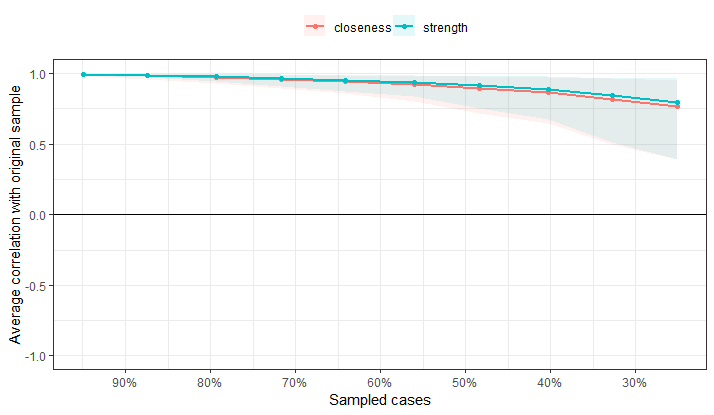


**Figure S11.** Centrality stability for depressive symptoms network in the high school boys.


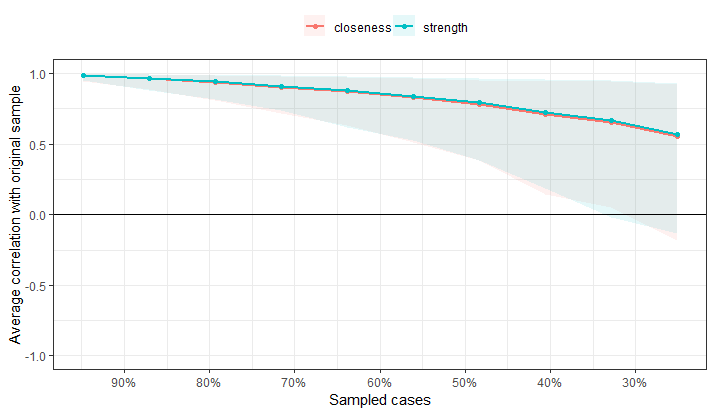


**Figure S12.** Centrality stability for depressive symptoms network in the high school girls.

**3. The edges with significant differences in strength**

**Table S1.** Edge invariance test between primary school boys and girls (p < 0.05)

| **Number** | **Var1** | **Var2** | **Absolute difference** | **p-value** |
| --- | --- | --- | --- | --- |
| 1 | PHQ-5 | PHQ-6 | 0.215 | 0.026 |
| 2 | PHQ-4 | PHQ-7 | 0.226 | 0.025 |
| 3 | PHQ-2 | PHQ-9 | 0.225 | 0.023 |
| 4 | PHQ-3 | PHQ-9 | 0.190 | 0.047 |
| 5 | PHQ-4 | PHQ-9 | 0.330 | 0.017 |
| 6 | PHQ-5 | PHQ-9 | 0.244 | 0.023 |
| 7 | PHQ-6 | PHQ-9 | 0.217 | 0.023 |
| 8 | PHQ-7 | PHQ-9 | 0.274 | 0.023 |
| 9 | PHQ-8 | PHQ-9 | 0.312 | 0.017 |

**Table S2.** Edge invariance test between high school boys and girls (p < 0.05)

| **Number** | **Var1** | **Var2** | **Absolute difference** | **p-value** |
| --- | --- | --- | --- | --- |
| 1 | PHQ-1 | PHQ-5 | 0.312 | 0.017 |
| 2 | PHQ-2 | PHQ-5 | 0.298 | 0.011 |
| 3 | PHQ-4 | PHQ-5 | 0.313 | 0.011 |
| 4 | PHQ-5 | PHQ-6 | 0.322 | 0.017 |
